# Cadmium Alters the Concentration of Fatty Acids in THP-1 Macrophages

**DOI:** 10.1007/s12011-017-1071-6

**Published:** 2017-06-10

**Authors:** Tomasz Olszowski, Izabela Gutowska, Irena Baranowska-Bosiacka, Agnieszka Łukomska, Arleta Drozd, Dariusz Chlubek

**Affiliations:** 1 Department of Hygiene and Epidemiology, Pomeranian Medical University, Powstańców Wlkp. 72 Str, 70-111 Szczecin, Poland; 2 Department of Biochemistry and Human Nutrition, Pomeranian Medical University, Broniewskiego 24 Str, 71-460 Szczecin, Poland; 3 Department of Biochemistry and Medical Chemistry, Pomeranian Medical University, Powstańców Wlkp. 72 Str, 70-111 Szczecin, Poland

**Keywords:** Cadmium, Fatty acids, THP-1 macrophages

## Abstract

Fatty acid composition of human immune cells influences their function. The aim of this study was to evaluate the effects of known toxicant and immunomodulator, cadmium, at low concentrations on levels of selected fatty acids (FAs) in THP-1 macrophages. The differentiation of THP-1 monocytes into macrophages was achieved by administration of phorbol myristate acetate. Macrophages were incubated with various cadmium chloride (CdCl_2_) solutions for 48 h at final concentrations of 5 nM, 20 nM, 200 nM, and 2 μM CdCl_2_. Fatty acids were extracted from samples according to the Folch method. The fatty acid levels were determined using gas chromatography. The following fatty acids were analyzed: long-chain saturated fatty acids (SFAs) palmitic acid and stearic acid, very long-chain saturated fatty acid (VLSFA) arachidic acid, monounsaturated fatty acids (MUFAs) palmitoleic acid, oleic acid and vaccenic acid, and *n*-6 polyunsaturated fatty acids (PUFAs) linoleic acid and arachidonic acid. Treatment of macrophages with very low concentrations of cadmium (5–200 nM) resulted in significant reduction in the levels of arachidic, palmitoleic, oleic, vaccenic, and linoleic acids and significant increase in arachidonic acid levels (following exposure to 5 nM Cd), without significant reduction of palmitic and stearic acid levels. Treatment of macrophages with the highest tested cadmium concentration (2 μM) produced significant reduction in the levels of all examined FAs: SFAs, VLSFA, MUFAs, and PUFAs. In conclusion, cadmium at tested concentrations caused significant alterations in THP-1 macrophage fatty acid levels, disrupting their composition, which might dysregulate fatty acid/lipid metabolism thus affecting macrophage behavior and inflammatory state.

## Introduction

Fatty acids (FAs) play multiple roles in immune cells, including serving as fuels for energy generation, contributing to physical and functional properties of cell membranes (as components of cell membrane phospholipids), or being covalent modifiers of protein structure. Fatty acids are also regulators of gene expression acting either on receptor activity, intracellular signaling, or transcription factor activation. Moreover, FAs are precursors for synthesis of bioactive lipid mediators, such as prostaglandins, leukotrienes, lipoxins, and resolvins [1]. Fatty acid composition of human immune cells influences their function via a variety of ways, including membrane alterations, effects on signal transduction pathways, or alterations in lipid mediators’ patterns [1].

A number of studies, both in vivo and in vitro, examined the effects of enrichment with different fatty acids on fatty acid profile in different cells, on cell function and behavior, and/or on different processes, such as inflammation [1–11].

Long-chain saturated fatty acids (SFAs), such as palmitic acid and stearic acid, have been found to produce a pro-inflammatory effect on several cell types, including human macrophages, inducing different inflammatory pathways [5, 9, 10].

Very long-chain saturated fatty acids (VLSFAs), such as arachidic acid, play an important role in normal physiology. They are components of sphingolipids, such as sphingomyelin and ceramides, and impart specific biological activities to the sphingolipids. There is growing evidence that circulating VLSFAs, in contrast to long-chain SFAs, may have beneficial biological properties [12].

The monounsaturated fatty acids (MUFAs) palmitoleic acid and oleic acid are the products of stearoyl-CoA desaturase (SCD), known also as Δ9-desaturase, the enzyme catalyzing biosynthesis of MUFAs from SFAs. The abovementioned MUFAs are the most abundant MUFAs of phospholipids, triglycerides, and cholesteryl esters. MUFAs may also act as mediators of signal transduction and cellular differentiation. Changes in SCD activity and in the balance between SFAs and MUFAs might have an effect in various diseases including cancer, diabetes, atherosclerosis, and obesity [13]. Oleic acid may have the ability to impede and reverse SFA-induced inflammation [11]. Oral administration of oleic acid decreased production of inflammatory mediators by rat macrophages [8].

Vaccenic acid is a ruminant-derived trans-fat and precursor of conjugated linoleic acid (CLA). Enrichment of diet of JCR:LA-cp rats (a rodent model of metabolic syndrome) with vaccenic acid has been shown to exert beneficial effects on lipids and lipoproteins [3]. In another study, vaccenic acid was demonstrated to decrease the pro-inflammatory markers IL-2 and TNF-α in this rat strain. However, such effect was not observed in normal rats, suggesting that the efficacy of vaccenic acid to benefit CVD might be more profound under disease conditions [4, 14].

The *n*-6 polyunsaturated fatty acid (PUFA) linoleic acid was demonstrated to blunt LPS-induced inflammation in THP-1 macrophages [2]. Oral administration of linoleic acid decreased the production of IL-1β, IL-6, IL-10, and VEGF in rat macrophages [8]. Linoleic acid can be converted to γ-linolenic acid and arachidonic acid. The *n*-6 PUFA arachidonic acid is the major substrate for the synthesis of eicosanoids, such as prostaglandins, leukotrienes, and other oxidized derivatives [15]. Enrichment of THP-1 macrophages with arachidonic acid was found to exert inhibitory effect on transcriptional levels of inflammatory factors and their secretion in culture media [6].

Cadmium is a well-known toxicant and immunomodulatory factor [16, 17], inducing inflammation in different biological systems [18–20]. It has been found that oxidative stress has been implicated in cadmium toxicity. Free radical induction by cadmium (i.e., superoxide anion, hydrogen peroxide, hydroxyl radical, and lipid radicals) appears to be a key factor responsible for its immunotoxic action [21]. Since cadmium is a redox-inactive metal, some indirect mechanisms, such as disruption of cellular antioxidant systems, inflammation, and involvement of iron for the Fenton reaction, must mediate free radical generation by cadmium [21]. In one of our previous studies, we examined one of the possible mechanisms involved in inflammatory reaction of THP-1 macrophages exposed to low concentrations of cadmium for 48 h: cadmium effects on COX-1 and COX-2 at messenger RNA (mRNA), protein, and enzymatic activity levels [22]. We demonstrated that cadmium at the highest tested concentration (2 μM) induced expression of COX-1 and COX-2 at the mRNA level, but not at the protein or enzymatic activity levels. In our another study, we observed increased ROS generation by all tested cadmium concentrations following 48-h exposure [23], which might contribute to COX-2 induction [24].

Several studies investigated the influence of cadmium on fatty acid and/or lipid metabolism in different experimental models [18, 19, 25–37]. The vast majority of those studies demonstrated significant alterations in fatty acid composition and/or fatty acid/lipid metabolism induced by cadmium. However, to the best of our knowledge, there was no study investigating the effects of cadmium on fatty acids in human immune cells. Cadmium-induced alterations in concentrations of fatty acids, such as arachidonic acid, being the precursors of products of cyclooxygenases activity (i.e., prostaglandin E2 and thromboxane B2), might play a key role in mechanisms of cadmium action in THP-1 macrophages described in our previous papers [22, 23]. Therefore, the aim of this study was to examine the effects of cadmium at low concentrations on the levels of selected fatty acids in THP-1 macrophages.

## Material and Methods

### Cell Culture and Treatment

The experiments were carried out on THP-1-derived macrophages. Human monocytic leukemia cell line THP-1 (American Type Culture Collection, Rockville, MD, USA) was selected, a widely used in vitro model for the investigation of the molecular mechanisms behind monocyte-to-macrophage differentiation [38]. THP-1 cells, seeded in an amount of about 2.5 × 10^6^ cells/well in 6-well plates, were induced to differentiate into macrophages by treatment with 100 nM phorbol myristate acetate (PMA) for 24 h. The next step was washing the adherent macrophages three times with PBS followed by 48-h culture (at 37 °C in 5% CO_2_) in medium containing 10% of FBS (fetal bovine serum; Gibco, Paisley, UK), penicillin (100 U/mL), streptomycin (100 mg/mL), and cadmium chloride (CdCl_2_). In this study, 5 nM, 20 nM, 200 nM, and 2 μM cadmium chloride concentrations were applied, since they corresponded to the cadmium level range detected in human blood due to environmental or occupational exposure [22, 39]. After incubation with cadmium, the cells were harvested by scraping and centrifuged (800×*g*, 10 min) to obtain pellets.

The trypan blue staining was used to determine the percent of viable cells using the Bright Line Hemacytometer (Sigma-Aldrich, Poznań, Polska). A cell cultures’ viability higher than 97% in the control group was the criterion of their use for experiments [40].

The experiments were conducted in six separate assays (each assay in three replicates).

### Extraction of Fatty Acids

The extraction of fatty acids from samples was performed according to the Folch method [41]. Fatty acids were protected against the oxidation reaction by the addition of 0.5% BHT solution to each sample. In a further step, the saponification of the samples was carried out with 1 mL of 2 M KOH methanolic solution at 70 °C for 20 min, followed by the methylation with 1 mL of 14% solution of boron trifluoride in methanol under the same conditions. Next, there was addition of 1 mL of *n*-hexane and 10 mL of saturated NaCl solution. After mixing, 1 mL of the *n*-hexane phase was collected for analysis.

### Analysis of Fatty Acids

Fatty acid concentrations were determined by gas chromatography using the Agilent Technologies 7890A GC System (SUPELCOWAX™ 10 Capillary GC Column (15 m × 0.10 mm, 0.10 μm)) (Supelco, Bellefonte, PA, USA). The following chromatographic conditions were applied: the initial temperature of 60 °C for 0 min, increased at a rate of 40 °C/min to 160 °C (0 min), increased at a rate of 30 °C/min to 190 °C (1 min), and then increased at a rate of 30 °C/min to 230 °C for 2.6 min, where it was maintained for 4.9 min. The total analysis took approximately 8 min. The gas flow rate was 0.8 mL/min; the carrier gas comprised of hydrogen. The identification of fatty acids was done by comparing their retention times with those of commercially available standards.

The fatty acid concentrations were determined based on standard curves and were expressed in milligrams per milliliter.

### Determination of Protein Concentration

Protein concentration was measured using a Micro BCA Protein Assay Kit (Thermo Scientific, Pierce Biotechnology, USA) and spectrophotometer (UVM340, ASYS). The bicinchoninic acid (BCA) protein assay was performed according to the manufacturer’s instructions [42].

### Statistical Analysis

For all quantitative parameters, the arithmetical means and the standard deviations (±SD) were presented. The normality of data for individual variables was assessed using the Shapiro-Wilk test. Since most of the distributions deviated from normality, the non-parametric tests were applied for further analyses. Wilcoxon matched-pairs test was applied to evaluate the differences between the concentrations of cadmium used. In order to assess the association between cadmium and fatty acid levels in THP-1 macrophages, Spearman’s correlation coefficients (*R*) and *p* values were calculated. The obtained results were processed using Statistica 10 (StatSoft, Poland) software. Levels of *p* < 0.05 were considered as statistically significant.

## Results

**Fig. 1:**
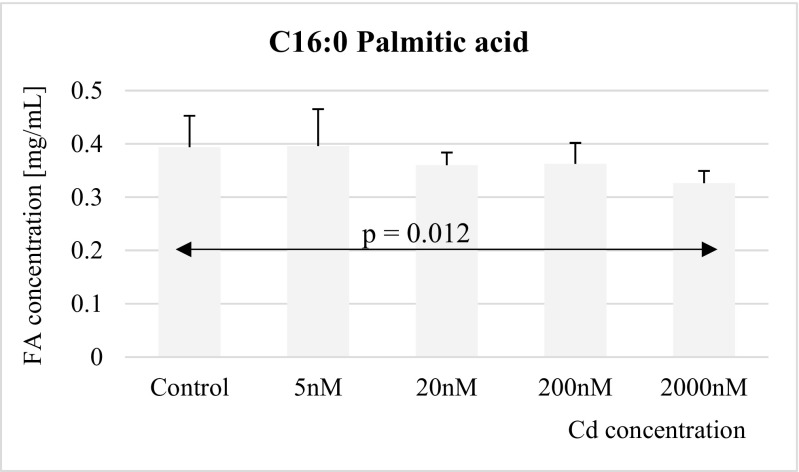
Influence of Cd on palmitic acid (C16:0) concentration in THP-1 macrophages

**Fig. 2:**
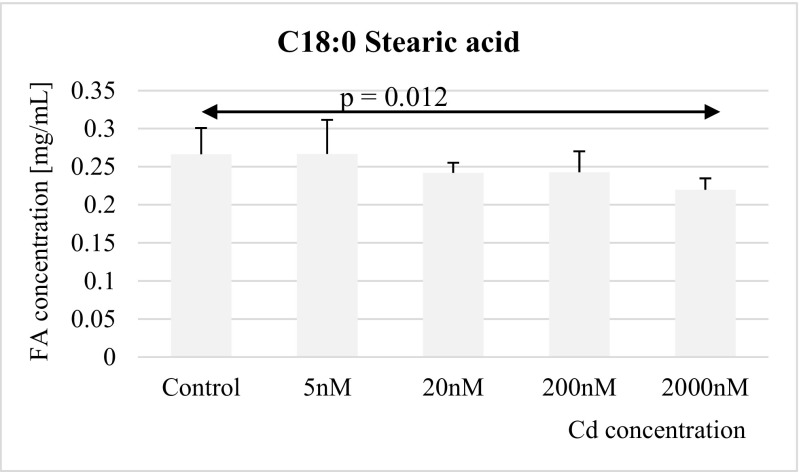
Influence of Cd on stearic acid (C18:0) concentration in THP-1 macrophages

**Fig. 3:**
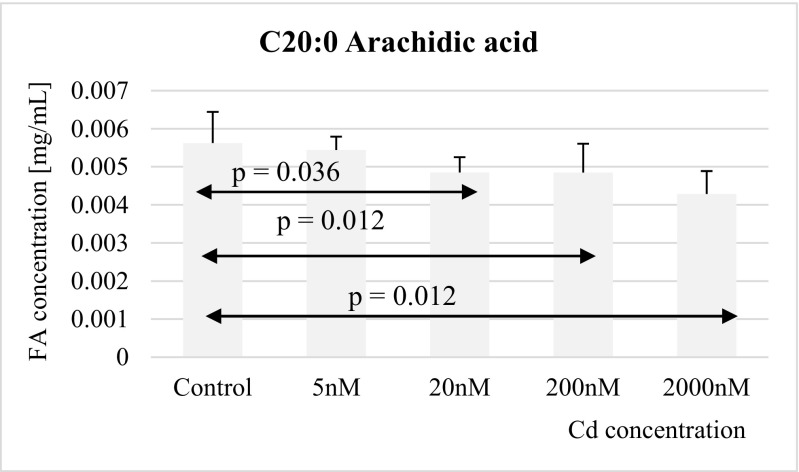
Influence of Cd on arachidic acid (C20:0) concentration in THP-1 macrophages

**Fig. 4:**
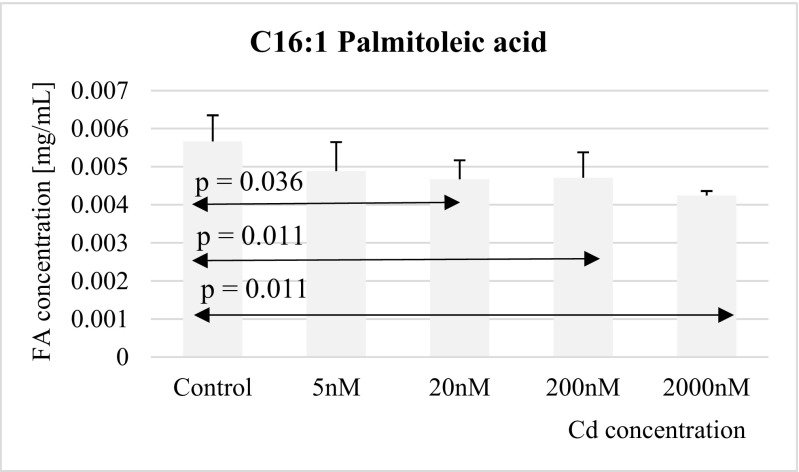
Influence of Cd on palmitoleic acid (C16:1) concentration in THP-1 macrophages

**Fig. 5:**
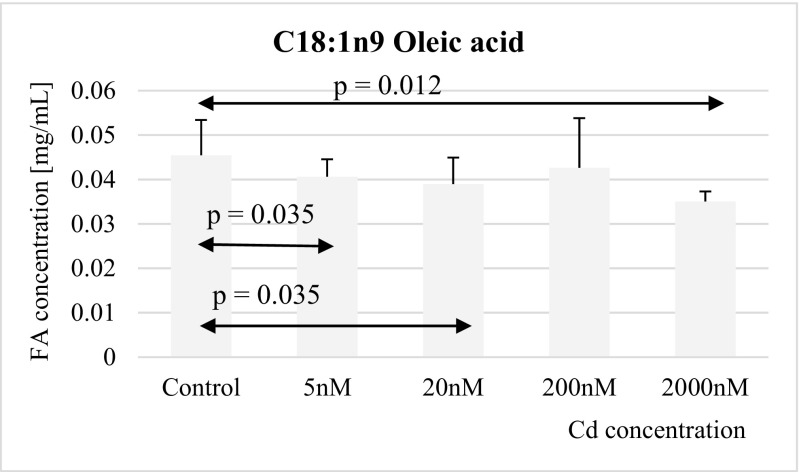
Influence of Cd on oleic acid (C18:1n9) concentration in THP-1 macrophages

**Fig. 6:**
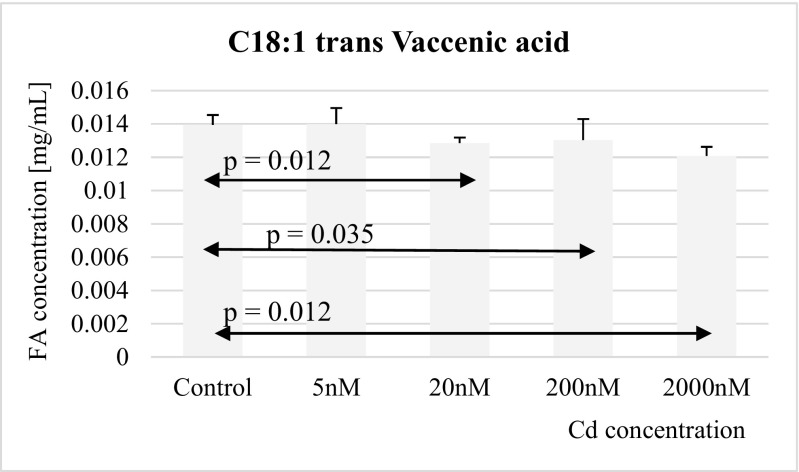
Influence of Cd on *trans* vaccenic acid (C18:1) concentration in THP-1 macrophages

**Fig. 7:**
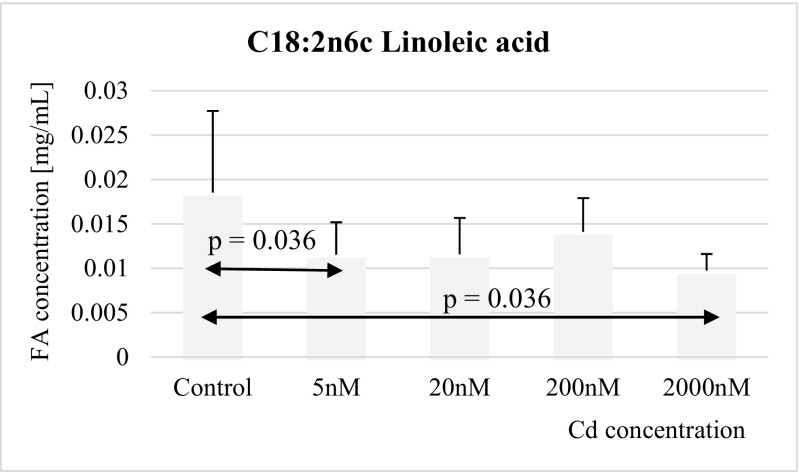
Influence of Cd on linoleic acid (C18:2n6c) concentration in THP-1 macrophages

**Fig. 8:**
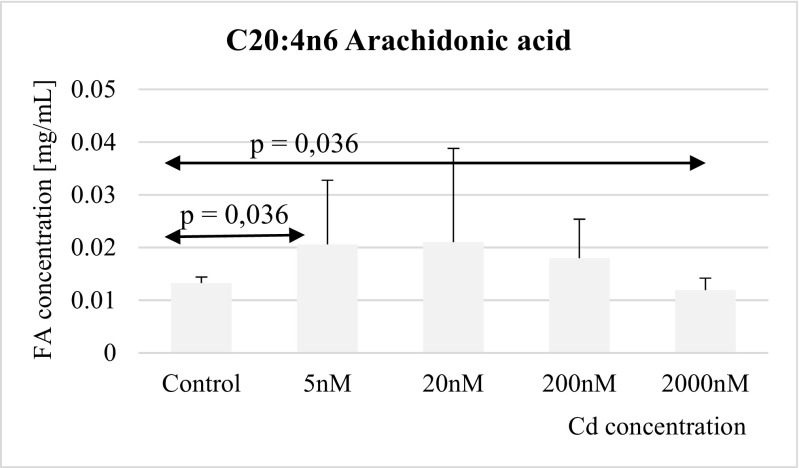
Influence of Cd on arachidonic acid (C20:4n6) concentration in THP-1 macrophages

The results of the study are presented in Figs. 1, 2, 3, 4, 5, 6, 7, and 8. Exposure to cadmium chloride at a concentration of 2 μM for 48 h caused significant reduction of concentration of palmitic acid in THP-1 macrophages. The exposure to lower tested cadmium concentrations (5, 20, and 200 nM) did not result in significant changes in palmitic acid concentrations, relative to controls (Fig. 1).

Similar effects of cadmium were found on stearic acid: 2 μM Cd resulted in significantly decreased, relative to controls, stearic acid concentration in macrophages (Fig. 2).

Cadmium at concentrations of 20 nM, 200 nM, and 2 μM markedly decreased the concentrations of arachidic acid in macrophages as compared to controls (Fig. 3).

THP-1 macrophages treated with 20 nM, 200 nM, and 2 μM Cd exhibited significantly lower (relative to controls) concentrations of palmitoleic acid (Fig. 4).

The other MUFA, oleic acid, concentration appeared to be significantly reduced in macrophages exposed to 5 nM, 20 nM, and 2 μM cadmium as compared to controls (Fig. 5).

The concentration of vaccenic acid was significantly reduced in macrophages cultured with 20 nM, 200 nM, and 2 μM cadmium chloride as compared to controls (Fig. 6).

Exposure to cadmium at concentrations 5 nM and 2 μM resulted in significant decrease in linoleic acid levels in THP-1 macrophages, as compared to controls (Fig. 7).

The effect of cadmium on arachidonic acid was biphasic depending on cadmium concentration: very low cadmium concentrations (i.e., 5 nM) appeared to increase markedly arachidonic acid levels, while the highest tested cadmium concentration (2 μM) resulted in significant reduction of arachidonic acid levels compared to control macrophage cultures (Fig. 8).

The analysis of correlation revealed negative correlation between the fatty acid content and cadmium concentration in the macrophage culture. Moderate negative correlation was found between cadmium and arachidic acid (*R* = −0.67; *p* = 0.00007) and between cadmium and vaccenic acid (*R* = −0.65; *p* = 0.0001), while low negative correlation was demonstrated between cadmium and stearic acid (*R* = −0.49; *p* = 0.007) and between cadmium and oleic acid (*R* = −0.48; *p* = 0.009).

## Discussion

Cadmium is a well-known immunomodulatory factor [16, 17]. This study is a continuation of our previous studies which main aim was to describe some biochemical and molecular mechanisms of toxicity of cadmium (at low concentrations) in THP-1 macrophages (the effects on COXs, ROS, and apoptosis) using the same experimental conditions (i.e., cadmium concentrations of 5 nM, 20 nM, 200 nM, and 2 μM, 48-h Cd exposure) [22, 23]. Mitochondria constitute important targets of cadmium toxicity [43]. The following sequence of events has been proposed to occur in mitochondria as a result of cadmium action: cadmium binding to protein thiols in mitochondrial membrane affecting mitochondrial permeability transition, inhibition of respiratory chain reaction, and then generation of ROS [44]. Cadmium was shown to cause disturbances in functioning of complex III of the electron transfer chain (i.e., inhibition of that complex) resulting in the increase in ROS generation [43, 45]. In one of our previous studies, we demonstrated decreased mitochondrial membrane potential and increased mitochondrial ROS generation in THP-1 macrophages cultured with all tested cadmium concentrations (5 nM, 20 nM, 200 nM, and 2 μM) for 48 h [23]. In the present study, we wanted to test whether cadmium modifies the fatty acid profile in THP-1 macrophages. We demonstrated that macrophages exposed to cadmium exhibited marked alterations in the levels of analyzed fatty acids. Treatment of macrophages with very low concentrations of cadmium (5–200 nM) resulted in significant reduction in the levels of arachidic, palmitoleic, oleic, vaccenic, and linoleic acids and significant increase in arachidonic acid levels (following exposure to 5 nM Cd), without significant reduction of palmitic and stearic acid levels. Treatment of macrophages with the highest tested cadmium concentration (2 μM) for 48 h produced significant reduction in the levels of all examined FAs: SFAs, VLSFA, MUFAs, and PUFAs.

Similar results were obtained by Steibert and Kokot in a different experimental model: the authors demonstrated that fatty acid biosynthesis was significantly inhibited in cytoplasmic fraction of hepatocytes of Wistar rats treated with 0.25 mg Cd/kg body wt. or higher doses for 20 days as compared to controls [46].

Regarding the effects of Cd on SFA concentrations, our study results seem to be discordant to the results of Ramirez and Gimenez [30], Larregle et al. [34], Konar et al. [36], and Kudo et al. [26], who demonstrated increased proportions of some SFAs due to cadmium treatment in different experimental systems. However, our study results seem to be in line with the results of Farid et al., who showed significant inhibitory effect of Cd on palmitate metabolism [25].

In our study, the effect of cadmium at most tested concentrations on MUFAs levels in macrophages was found to be inhibitory. Such result is in accordance with the findings of Kudo and Waku [28], Kudo and coworkers [47] and Ramirez and Gimenez [30], but in disagreement with the findings of Larregle and coworkers [34].

According to literature, cadmium was found to decrease MUFA:SFA ratio [30, 34], which reflects the cadmium-induced inhibition of Δ9-desaturase [26, 28, 47]. We speculate that the lower tested cadmium concentrations might decrease MUFA:SFA ratio, because MUFA concentrations were significantly reduced without significant alteration of SFA concentrations. Taking this into consideration, cadmium might reduce Δ9-desaturase activity in our experimental model. Moreover, the results of correlation analysis might support this hypothesis: the inverse correlation between cadmium and stearic acid (the substrate of SCD enzyme) and between cadmium and oleic acid (the product of SCD enzyme).

Cadmium-disturbed MUFA:SFA ratio might influence different aspects of cell metabolism, and, for example, promote inflammation [48].

Exposure of THP-1 macrophages to 5 nM and 2 μM Cd caused significant reduction of levels of linoleic acid. Such result is consistent with the study results of Larregle et al. [34] and Ramirez and Gimenez [30]. Significantly decreased concentration of linoleic acid in macrophages exposed to 5 nM Cd could be due to an increase of its conversion to arachidonic acid, which is catalyzed by delta-6 desaturase, since the concentration of arachidonic acid was significantly elevated by this cadmium concentration.

Significantly increased arachidonic acid levels in macrophages following exposure to the lowest tested cadmium concentration (5 nM) might be explained by cadmium-induced, diminished utilization of arachidonic acid by COXs as a substrate to prostaglandin and thromboxane synthesis. The results obtained in our previous study, i.e., the lack of significant alterations in mRNA and protein expression as well as enzymatic activity of COXs in THP-1 macrophages exposed to 5 nM Cd for 48 h [22], seem to support the above supposition.

Another possible explanation for the increased arachidonic acid concentration by 5 nM Cd might be cadmium-induced increased activity of delta-6 desaturase, catalyzing conversion of linoleic acid to arachidonic acid [28].

Treatment of THP-1 macrophages with 2 μM cadmium resulted in significant decrease in the levels of arachidonic acid. Such result is consistent with the results obtained by Kudo et al. [27], but it is opposite the findings of Ramirez and coworkers [29], Ramirez and Gimenez [30], and Larregle and coworkers [34].

The decreased concentrations of MUFAs and PUFAs by 2 μM Cd may be due to metal induction of the cyclooxygenase pathway [49]. However, in one of our previous studies using the same experimental model and experimental conditions as in the present study, we demonstrated that cadmium at this concentration (2 μM) induced expression of COX-1 and COX-2 only at the mRNA level, but not at protein or enzymatic activity levels [22]. Therefore, one may speculate that other than COX inflammatory pathway(s) might be induced by such cadmium concentrations; for example, lipoxygenase and its product, leukotriene B_4_ (LTB_4_) [18, 49].

It is also possible that the reduction in fatty acid levels by the highest tested cadmium concentration (i.e., 2 μM) might have been due to cell toxicity of cadmium, since in one of our previous studies (employing the same experimental model and experimental conditions), we found significant reduction in THP-1 macrophages viability and increased cell death by apoptosis and necrosis at this cadmium concentration [23]. Maybe the decreased fatty acid concentrations by 2 μM Cd might be the consequence of cadmium-induced, lowered cellular metabolism, and especially, the dysregulated fatty acid/lipid metabolism [50, 51].

In the present study, the degrees of reduction in fatty acid concentrations by cadmium were small (although statistically significant). However, in our opinion, even small alterations in fatty acid levels induced by cadmium (for example, altered MUFA:SFA ratio, altered arachidonic acid level, or altered, disturbed membrane fluidity) might have biological significance. For example, altered MUFA:SFA ratio and altered concentration of PUFA arachidonic acid might have an effect on inflammation [6, 48]. According to Garcia et al., even slight changes in membrane fluidity may result in aberrant numerous cell functions (e.g., signal transduction or membrane-associated enzymatic activities), leading to some pathological processes [52]. For example, decreased fluidity was demonstrated in erythrocyte ghosts and placental membranes treated with chloride salts of cadmium, mercury, and lead [53, 54]. It is possible that cadmium-induced altered membrane fluidity might affect macrophage adhesion and phagocytic activity [55].

Moreover, despite the fact that cadmium-induced reductions in fatty acid levels were small, the cumulative effect of cadmium after long exposure periods may not be excluded.

In conclusion, cadmium at tested concentrations caused significant alterations in THP-1 macrophage fatty acid levels, disrupting their composition, which might dysregulate fatty acid/lipid metabolism, thus affecting macrophage behavior and inflammatory state.
